# Testicular Torsion: Preliminary Results of In Vitro Cell Stimulation Using Chorionic Gonadotropin

**DOI:** 10.3390/cells11030450

**Published:** 2022-01-28

**Authors:** Andrea Errico, Francesco Saverio Camoglio, Nicola Zampieri, Ilaria Dando

**Affiliations:** 1 Department of Neurosciences, Biomedicine and Movement Sciences, University of Verona, 37134 Verona, Italy; andrea.errico@univr.it; 2 Department of Surgery, Dentistry, Paediatrics and Gynaecology, Paediatric Fertility Lab, Woman and Child Hospital, Division of Pediatric Surgery, University of Verona, 37134 Verona, Italy; francesco.camoglio@univr.it

**Keywords:** human chorionic gonadotropin, gonadotropins, testicular torsion, gubernaculum testis, cell proliferation, hormonal therapy

## Abstract

Testicular torsion is a pathology that occurs in young males generally before the age of 25. Despite surgery representing the only effective approach, there is still a need to identify a marker that can predict whether a preserved testicle will be functional. In addition, no therapeutic approach is currently considered in the post-operative phase. Through an approach based on the in vitro culture of a tissue strictly linked to the testicle, the gubernaculum, we defined the healthy state of the organ and the possible responsiveness to a therapy used in the andrology field, chorionic gonadotropin (hCG). Firstly, we optimized a protocol to obtain viable cells starting from a small piece of gubernacular tissue harvested during surgery with the aim to amplify cells in vitro. Intriguingly, only for a patient whose testicle had been removed during surgery due to an excessive necrotic area, gubernacular cells were not able to grow in culture. These data support the possibility of exploiting the gubernaculum to evaluate the healthy state of the testicle. Then, as we demonstrate that gubernacular cells express a luteinizing hormone receptor, to which hCG is specific, we analyzed the cellular response to hCG treatment on in vitro cultured cells derived from patients affected by testicular torsion. Our study opens the way for the possibility of evaluating testicle wellbeing after derotation through in vitro culture of a small piece of gubernaculum together with predicting the response to the treatment with hCG, which can have a positive effect on cell proliferation and vascularization.

## 1. Introduction

Testicular and spermatic cord torsion are relatively common, representing a surgical emergency. This pathology occurs in 1 out of 4000 males younger than 25 years [1]. It accounts for approximately 10% to 15% of acute scrotal disease in children and results in an orchiectomy rate of 42% in boys undergoing surgery for testicular torsion [1]. This event generally takes place antenatally/in the early postnatal period as an entire cord twisting or in older children and adults as a twisting of the cord within the tunica vaginalis [2]. In both situations, the first phase of spermatic cord twist is characterized by the increase in venous pressure and congestion, followed by a second phase in which there is a decrease in arterial blood flow with ischemia. However, a wide range of diverse events may induce acute scrotum pain or dysfunction, such as epididymo-orchitis, idiopathic scrotal edema, infection, torsion of the appendix testicle or appendix epididymis, torsion of the spermatic cord, trauma, tumor, and varicocele [2]. Therefore, despite most patients not requiring urgent intervention, a significant minority present testicular torsion. However, in the case of testicular torsion, the viability of the testis decreases 6 h after the onset of symptoms, which necessarily requires a prompt diagnosis. The risk of testicular loss or unnecessary surgery calls for the necessity of novel diagnostic techniques; for instance, a recent study focused on the role of mean platelet volume in the diagnosis of testicular torsion [1]. By now, the only safe approach to resolve testicular torsion is surgery, which should be performed as soon as possible, and testicular viability is secondary to both the number of spermatic cord turns and the post-derotation recovery. Testis vascularization originates from the spermatic cord as well as from the cremasteric artery and gubernaculum. In fact, during testicular torsion, the third path of blood supply to the testis, i.e., the gubernaculum, is generally twisted and ischemic. Real therapeutic doubt arises from the ability to predict which testes left in place after derotation will be viable and functional in the long term.

The gubernaculum testis is an elongated conical structure composed of loose extracellular matrix and mesenchymal cells, mainly including fibroblasts and muscle cells, which have the function to pull the testes through the abdomen and the inguinal canal down into the scrotum. From a histological examination, it has been reported that the gubernaculum consists of moderately vascular, fibrofatty tissue with a core of essentially longitudinally oriented collagenous fibers surrounded by mature fatty tissue [3]. Insulin-like 3 peptide (INSL3) is a hormone produced by the gonads and is the ligand of the relaxin family peptide receptor 2 (RXFP2), a G protein-coupled receptor (GPCR) [4]. In males, INSL3 is primarily produced by Leydig cells, and RXFP2 has been shown to be highly expressed in the gubernaculum. During embryogenesis, testicular descent consists of two phases: the transabdominal and inguinoscrotal phases. The INSL3/RXFP2 axis has an essential role in the development of the gubernaculum for the initial transabdominal descent of the testis and in the maintenance of reproductive health in men [5,6]. Despite the inguinoscrotal phase being mainly mediated by androgens, some studies have also shown the involvement of INSL3/RXFP2 [6]. In addition, it has been shown that rat gubernacular cells show an increased production of cAMP upon stimulation with INSL3 and that bilateral cryptorchidism in INSL3- and RXFP2-deficient mice is linked with the impaired development of the gubernaculum [4]. During testicular migration, the gubernaculum undergoes extensive remodeling, which contributes to the testicular descent toward the scrotum. A study showed that during 15 to 16 weeks of gestation, collagen fibers that were composed of the gubernaculum were sparser and more embedded in a loose extracellular matrix; then, at 28 weeks of gestation, the number of fibers gradually increased, making the gubernaculum mostly collagenous. Fibroblasts largely predominate over other cell types and decrease in number with gestational age, whereas smooth muscle cells are restricted to the walls of blood vessels. Striated muscle cells are generally detected at the scrotal end of the gubernaculum, decreasing in number with age [7]. Usually, after birth, the muscles and connective tissues obliterate any opening and the gubernaculum testis becomes the scrotal ligament (SL), persisting in adults as a fibrous residual structure only individualized as microscopic connective fiber. The SL is typically described as a ‘‘Y-shape’’ ligament with the testis and epididymis as proximal insertions and the scrotum as a distal attachment. However, in some cases, authors have shown the absence of attachment to the adult scrotum, supporting the involution of the gubernaculum after birth and therefore the possible absence of SL [8].

Human chorionic gonadotropin (hCG) is a glycoprotein hormone that belongs to the gonadotropin hormone family, which also includes the luteinizing hormone (LH) and the follicle-stimulating hormone (FSH) and binds to the LH receptor (LHR). HCG is clinically used in post-operative patients, more specifically cryptorchid patients, to achieve higher testicular volume and function [9].

Interestingly, it has been reported that, when treated with hCG, gubernaculum components alter significantly and become rich in vessels [10].

Although the positive effect of hCG treatment on the gubernaculum in cryptorchid patients is partially known, its clinical use in other andrological pathologies, including testicular torsion, has not been applied. Thus, with this study, we show that gubernacular tissues that have been derived from patients affected by testicular torsion can be cultured and amplified in vitro in order to test the efficiency of hCG therapy, which can also be used in this pathology to recover testicle functionality. In addition, the union of biological and clinical approaches may help in the identification of a best personalized posology prior to administration to the patient.

## 2. Materials and Methods

This prospective observational study was approved by the Pediatric Fertility Lab internal review board (09/2020).

The Ethical Committee of “Azienda Ospedaliera Universitaria Integrata” (AOUI) of Verona, Italy, had already approved the program “Fertility Potential (FePo) 2.0” (N. 3072 CESC), regarding the study of fertility preservation in pediatric and adolescent patients, this being the basis of the study aimed at the improvement of fertility potential in males.

Since May 2019, at our Fertility Lab Tissue Biobank, we have been collecting scrotal fat and testicular and gubernacular tissue from pediatric and adult patients operated on for testicular pathologies. Each patient is cataloged with a progressive number and identification. The tissue database is composed of samples from patients aged between 1 month and 50 years.

### 2.1. Patients

Informed consent was obtained from all the parents and patient compliance was 100%. The internal IRB approved the study. At our center, we collect samples of patients with primary azoospermia, patients with genetic syndromes associated with azoospermia, patients with undescended testis, patients with neoplasms, and patients with testicular neoplasms. In addition, patients with testicular torsion or testicular trauma are included.

### 2.2. Inclusion Criteria

We considered all patients with testicular torsion that were clinically diagnosed and treated within 24 h of symptom onset and surgically treated within 2 h after hospital arrival in order to avoid bias related to hospital coordination.

### 2.3. Exclusion Criteria

We excluded patients treated for torsion that had definitely occurred at least 24 h before symptom onset, either reported in history or showing an absence of pain on palpation. Moreover, patients reporting testicular trauma immediately before a suspected torsion or this being considered as the main cause of their torsion were excluded.

### 2.4. Surgical Procedure

All patients underwent surgery by the same operator under general anesthesia. All procedures were performed with a trans-scrotal incision. All testes were delivered, the torsion was resolved, and then 3 micro-biopsies were performed to verify the vitality. If necessary (no bleeding), the testes were removed. In cases with partial testicular viability (bleeding and/or good parenchymal color), the testes were left in place. All patients underwent gubernaculum biopsies to deduce more information about vascularization. No other procedures were performed on the contralateral testes.

### 2.5. Drugs and Chemicals

Highly purified human chorionic gonadotropin (hCG) extracted from pregnant women’s urine was kindly provided by IBSA Italia. As reported in the results section, we treated the cells in vitro with 100 UI/mL hCG once a week for 4 weeks and then analyzed cell proliferation after the end of the 5th week. We chose this therapeutic scheme because it was very similar to the scheme used in clinics for certain pediatric testicular pathologies. This hormonal treatment is also suggested in adulthood for infertility with different dosages.

### 2.6. Gubernacular Tissue Processing and In Vitro Culture

The gubernacula were processed by firstly cutting the piece of tissue (about 10–20 mm^3^) with a scalpel in 500 μL of phosphate-buffered saline (PBS) on a Petri dish in sterility under the biological hood. Then, the fragmented tissues were enzymatically disaggregated with 1X collagenase type I and hyaluronidase (both from Sigma-Aldrich, St. Louis, MO, USA, Merck) resuspended in 200 μL of a complete cell culture medium and incubated at 37 °C for 1 h, or until complete homogenization of the tissue. Cell viability was evaluated with a trypan blue assay and was variably around 20–40%. Finally, the cell suspension was grown in DMEM-Glutamax supplemented with 10% fetal bovine serum (FBS), 4.5 g/L glucose, and 50 μg/mL gentamicin sulfate (all from Gibco/Life Technologies, Waltham, MA, USA) and maintained at 37 °C with 5% CO_2_ in Corning Primaria cell culture flasks (Corning, New York, NY, USA).

Before hCG treatment, cells were maintained for 16 h in an FBS-free medium as FBS generally contains FSH and LH at different concentrations from lot-to-lot, as reported by different manufacturers’ sheets. Then, cells were treated with the indicated doses and for the indicated time (see figure legends) with hCG by keeping cells in the medium without FBS.

Bright field cell images were acquired with an inverted microscope (Axio Vert. A1, Zeiss, Oberkochen, Germany).

### 2.7. RNA Extraction and qPCR

RNA extraction and real-time quantitative PCR (qPCR) were performed as previously described [11]. The positive control cells were MCF7 (breast cancer cell line) and the negative control cells were PaCa44 (pancreatic cancer cell line), as previously described [12]. Briefly, total RNA was extracted from 1 × 10^5^ cells using a Single Cell RNA Purification Kit (Norgen Biotek, Thorold, ON, Canada) and 0.5 μg of RNA was reverse-transcribed using first-strand cDNA synthesis. The quality of RNA was evaluated by running 0.5 μg of RNA for each sample on an agarose gel. Real-time quantification was performed in triplicate samples by SYBR-Green detection chemistry with the GoTaq qPCR Master Mix (Promega, Madison, WI, USA) on a QuantStudio 3 Real-Time PCR System (Thermo Fisher Scientific, Waltham, MA, USA). The primers used were: *LHR* forward, 5′-GCTGCGATTAAGACATGCCA-3′, *LHR* reverse, 5′-AGAAGGCCACCACATTGAGA-3′; Androgen Receptor (*AR*) forward, 5′-CCCACTTGTGTCAAAAGCGA-3′, *AR* reverse, 5′-GCAGCTTCCACATGTGAGAG-3′; and Ribosomal Protein Lateral Stalk Subunit P0 (*RPLP0*) forward, 5’-ACATGTTGCTGGCCAATAAGGT-3’, *RPLP0* reverse, 5’-CCTAAAGCCTGGAAAAAGGAGG-3’. The negative control samples were without cDNA. The cycling conditions used were: 95 °C for 10 min, 40 cycles at 95 °C for 15 s, 60 °C for 1 min, 95 °C for 15 s, and 60 °C for 15 s. The average cycle threshold of each triplicate was analyzed according to the 2^−^^ΔΔ^^Ct^ method. *RPLP0* gene expression was used as an endogenous control to standardize mRNA expression. All reactions were performed in three independent experiments.

### 2.8. Cell Proliferation Assay

Gubernacular cells were plated in 96-well cell culture plates (5 × 10^3^ cells/well) and incubated at 37 °C with 5% CO_2_. Cell viability was measured by crystal violet assay (Merck Millipore) according to the manufacturer’s protocol, and absorbance was measured by spectrophotometric analysis (A595 nm). The crystal violet assay is designed to work with adherent cells, and when cells die, they detach from the surface of the plate. Thus, this assay is suitable for in vitro cell proliferation and cell cytotoxicity studies, as reported in several manufacturers’ sheets. At each treatment time point, we added 25 mL of medium containing solvent or hCG to the control or treated cells, respectively, in the wells. Gubernacular cells derived from patients affected by cryptorchidism with normal testicular viability were used as a control. Three independent biological replicates were performed, each in triplicate.

### 2.9. Statistical Analysis

ANOVA (post hoc Tukey) analysis by GraphPad Prism 5 (GraphPad Software, Inc., San Diego, CA, USA) or Student’s *t*-test (two-tailed) were conducted. The *p*-values < 0.05, 0.01, or 0.001 were considered significantly different (for details, see figure legends).

## 3. Results

### 3.1. Clinical Retrospective Evaluation

During the study period, five patients were treated for testicular torsion who were aged between 12 and 15 years.

Of these patients, two were excluded because their symptoms and torsion had occurred more than 24 h after hospital arrival. Therefore, we considered three patients, two of whom had preserved testicles and one for whom the testicle was removed. In detail:

Patient #4: An adolescent 12-year-old male with testicular torsion with an onset of symptoms less than 8 h before surgery. Spermatic cord torsion: two loops (720°). Testis left in place.

Patient #12: An adolescent 14-year-old male with testicular torsion with an onset of symptoms less than 14 h before surgery. Spermatic cord torsion: one loop and half. Testis removed.

Patient #15: An adolescent 14-year-old male with testicular torsion with an onset of symptoms less than 10 h before surgery. Spermatic cord torsion: one loop and half. Testis left in place.

### 3.2. Gubernacular Tissue Processing and In Vitro Cell Growth

**Figure 1:**
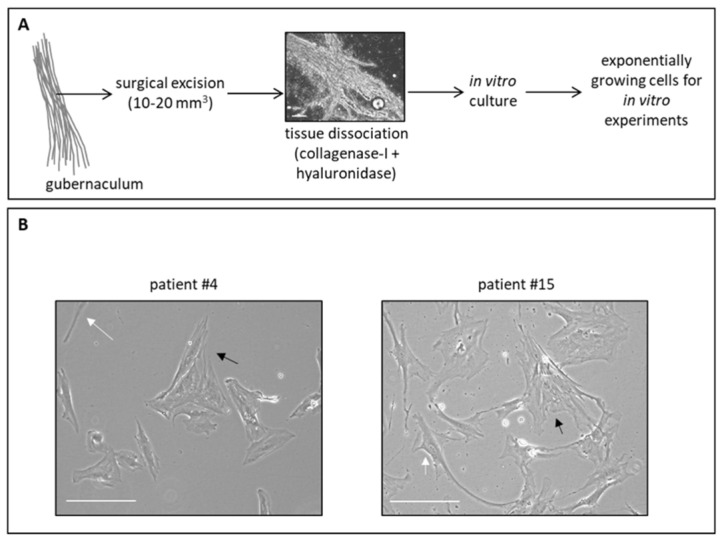
Gubernacular tissue in vitro culture. (**A**) Schematic representation of the protocol that we optimized to isolate and in vitro cultivate gubernacular cells. Scale bar: 100 μm. (**B**) Representative, bright field images of gubernacular cells of patients #4 and #15. White arrows indicate cells with fibroblast features and black arrows indicate cells with smooth muscle features. Scale bar: 25 μm.

The gubernaculum biopsies obtained from pediatric patients were generally about 10–20 mm^3^ in volume. The gubernacula of patients #4, #12, and #15 were processed as fresh or thawed tissue; the latter was previously stored at −80°C for one week and was stored in liquid nitrogen after that. As reported in Figure 1A, the gubernacular tissue was disaggregated by using an enzymatic mix composed of 1X collagenase-I and hyaluronidase and was incubated for at least 1 h at 37 °C or until the tissue appeared as a homogenous solution. Then, the cells obtained from the processed tissue were counted; cell number was estimated to be at least more than 300,000 for each patient. Then, the complete cell suspensions were grown in a T25 culture flask. Interestingly, the lag period during which cells adapted to the in vitro condition was variable among patients. We considered the healthy state of the cells, based on cellular morphology, and the gain of about 80–90% of confluence as signs of exponentially growing cells. As reported in Table 1, it is evident that the temporal window within which cells started to exponentially grow was different among patients. Indeed, patient #4 derived gubernacular cells that quickly attached to the flask, that presented a healthy cellular state, and that started to exponentially grow 7 days after tissue processing (Table 1). Conversely, patient #15 derived gubernacular cells that were struggling to attach to the flask and only after 15 days presented a healthy cellular status and started to exponentially grow 30 days after tissue processing (Table 1). Finally, for patient #12, cells were never able to attach to the flask and grow (not applicable: N/A; Table 1). For patients #4 and #15, exponentially growing cells were harvested to perform experiments or were divided into two other flasks to amplify the cells. In both patients, cells were monitored during the culture period, which was about 120 days; after this period, cells entered a senescent status and slowed down their proliferation.

As represented in Figure 1B, the cellular morphology of gubernacular cells is composed of a mix of cell types, with some cells showing a fibroblast shape (white arrows) and other cells presenting a bigger size with some striae, typical of smooth and striated muscle cells containing actin (black arrows).

### 3.3. Gubernacular Cells Express AR and LHR

**Figure 2:**
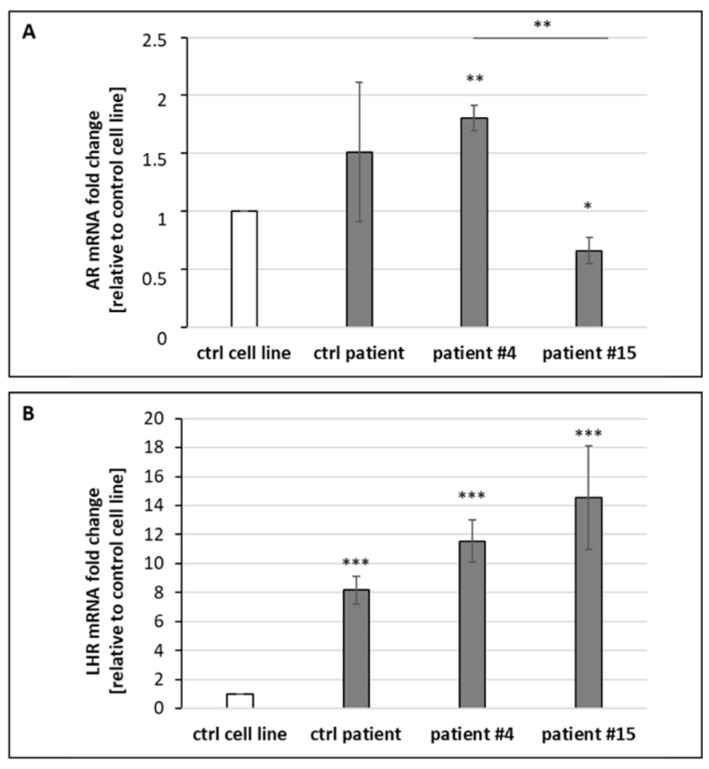
mRNA expression levels of *AR* and *LHR* in gubernacular cells. Analysis of mRNA expression levels of (**A**) *AR* and (**B**) *LHR* in gubernacular cells cultured in vitro. Receptor mRNA levels were normalized on the housekeeping gene (*RPLP0*) expression and reported as fold change relative to the breast cancer cell line MCF7. PaCa44 cell line, used as a negative control, was not reported in the analysis because, in these cells, the expression of the two receptors was absent (undetermined). Gubernacular cells derived from a 2-year-old patient affected by cryptorchidism were used as a control (control “ctrl” patient). Values are the means (±SD) of two independent biological replicates. Statistical legend: *p* < 0.05 (*), *p* < 0.01 (**), and *p* < 0.001 (***) for gubernacular cells versus control cells (MCF7) or for patient #4 versus patient #15.

The expression and role of the androgen receptor (AR) in gubernaculum testis have been deeply investigated during testicular descent alongside the demonstration that AR signaling in gubernacular cells is required for its eversion and outgrowth [13]. However, to our knowledge, there is no evidence about the expression of AR in the gubernaculum of patients affected by testicular torsion at pubertal age or either about the expression of another important receptor that has a key role in testicular function, i.e., the luteinizing hormone receptor (LHR). Thus, we quantitatively analyzed the mRNA levels of *AR* and *LHR* in gubernacular cells derived from the control patient (i.e., the patient affected by cryptorchidism) and patients #4 and #15. We used a breast cancer cell line (MCF7) for the positive control cells and a pancreatic cancer cell line (PaCa44) for the negative control cells, whose values were undetectable, as previously described [12]. The data reported in Figure 2A show that *AR* expression was differentially expressed among the two patients. Indeed, patient #4 had a significantly higher *AR* mRNA expression relative to the control cells and to patient #15, whereas the latter showed an opposite trend. The control patient showed an *AR* mRNA expression similar to that of the control cell line. In regards to *LHR*, patients #4 and #15 and the control patient showed a similar level of *LHR* mRNA induction in comparison to the control cells, bearing a significantly higher expression (Figure 2B).

### 3.4. Gubernacular Cell Proliferation Is Stimulated by hCG Treatment

**Figure 3:**
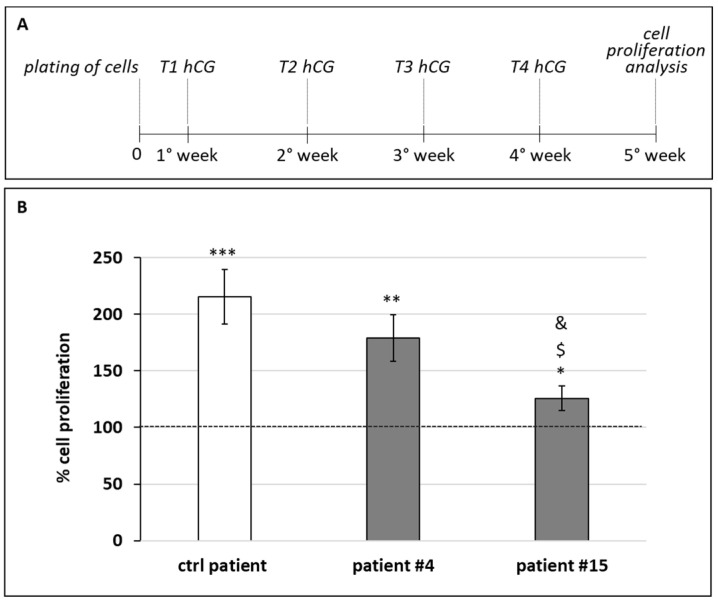
Effect of hCG on gubernacular cell proliferation. (**A**) Schematic representation of the treatment protocol adopted to analyze the effect of hCG on cell proliferation. (**B**) Cell proliferation of gubernacular cells cultured at 37 °C with 5% CO_2_ in an FBS-deprived medium treated with 100 UI/mL hCG once a week for 4 weeks. Gubernacular cells derived from a 2-year-old patient affected by cryptorchidism were used as a control. The percentage of cell proliferation was evaluated by analyzing the data of hCG treated cells for a specific patient (corresponding to the histograms) with the data obtained for the same patient cells that were not treated whose proliferation corresponded to 100% (dashed line). Cell proliferation was measured by crystal violet assay and absorbance was measured by spectrophotometric analysis (A595 nm). Values are the means (±SD) of three independent biological replicates. Statistical legend: *p* < 0.5 (*), *p* < 0.01 (**), and *p* < 0.001 (***) for treated cells versus untreated cells of the same patient; *p* < 0.05 ($) for hCG treated cells of patient #15 versus hCG treated cells of control patient; *p* < 0.05 (&) for hCG treated cells of patient #4 versus hCG treated cells of patient #15.

As our data revealed that the gubernaculum also expresses LHR (Figure 2B), which is the receptor of LH and hCG, we tested the in vitro response of the gubernacular cells of patients #4 and #15 to hCG treatment by analyzing cell proliferation. As schematically represented in Figure 3A, we treated the cells in vitro with 100 UI/mL hCG once a week for 4 weeks and then analyzed cell proliferation after the end of the 5th week. We chose this therapeutic scheme because it was very similar to the posology used in clinics for certain testicular pathologies. It is noteworthy that, as reported in the Materials and Methods, cells were treated and maintained in culture in a medium depleted with FBS, which included LH and FSH [12], in order to evaluate the effect on cell proliferation determined only by hCG. As a control, we used gubernacular cells derived from a younger patient (2 years old) who had not been affected by testicular torsion but by cryptorchidism, here reported as the “control patient” (Figure 3B). Our data showed that both patients #4 and #15, as well as the control patient, showed a significant increase in the percentage of cell proliferation when treated in vitro with hCG in comparison to the respective control cells, i.e., the same patient cells but untreated, thus corresponding to 100% of cell proliferation (Figure 3B). In addition, the gubernacular cells of patient #15 treated with hCG showed a less proliferative capacity in comparison to both patient #4 and the control patient.

## 4. Discussion

Testicular torsion is a disease that necessarily requires a prompt diagnosis and treatment alongside a surgeon’s decision on whether to preserve the testes or not. A negative long-term effect of a damaged testicle left in place could be an alteration of the fertility potential of the subject. At present, the only safe approach to treat testicular torsion is timely surgery, albeit no adjuvant therapeutic approach is currently considered in the post-operative phase. As chorionic gonadotropin is a treatment generally used to improve fertility potential in infertile men and increase testicular trophism in post-operative cryptorchid patients, we hypothesized that the administration of this treatment in patients with testicular torsion may improve testicle health. Starting from this assumption, our study focused on the in vitro validation of these observations in order to understand if this approach could also be leveraged in this therapeutic field. In particular, we focused our attention on the gubernaculum because it is known to have an important role not only during testicle descent but also as one of the major sources of testis vascularization. Indeed, it has been demonstrated that gubernacular components become rich in vessels when treated with gonadotropin [10]. In addition, during testicular torsion, the gubernaculum is generally twisted and ischemic, thus representing an important element that can be exploited to analyze the viability of the testicle and the possible response to the treatment with chorionic gonadotropin.

To accomplish the aims of this study, we first optimized a protocol to culture and amplify in vitro gubernacular cells. The potentiality to use gubernaculum for these in vitro studies was twofold: first, the testicle, which is suffering due to torsion, could be completely preserved and surgically untouched; second, from our preliminary data (not shown), the culture of gubernacular tissue provides a higher yield of in vitro growing cells in comparison to testicular cells, which propagate more slowly. Indeed, we demonstrated that the disruption of a small portion of gubernacular tissue harvested during surgery in pediatric patients allows the obtainment of viable primary cells that possess the ability to grow in culture. However, as we also obtained tissue from a patient who underwent orchiectomy, here, we revealed that there was a good correlation between the clinical evaluation of testicle health and the ability of gubernacular cells to grow in vitro; indeed, the isolated cells derived from patient #12, whose testicle had been removed, were not able to grow in vitro, in line with the surgeon’s decision to perform orchiectomy due to the presence of a necrotic testicle. Thus, this initial evidence suggests that the in vitro culture of the gubernaculum may be exploited as an evaluation of the state of the testicle, evidencing the viability of the organ and thereafter confirming to the surgeon whether there is a necessity or not to remove a testicle that showed a border-line health appearance.

Further evidence that emerged from our data was that gubernacular cells not only expressed the androgen receptor (*AR*) but also the luteinizing hormone receptor (*LHR*), which is essential to assess a possible response to gonadotropin, being its specific receptor. It is interesting to highlight that, for the control patient, who was affected by cryptorchidism, there was an expression of high levels *LHR* mRNA, further supporting that the gubernaculum is a candidate tissue for the response to hCG therapy. Afterward, we treated gubernacular cells derived from patients #4 and #15 with a dosage and timing of treatment that was as close as possible to the posology that is provided to patients. Indeed, we treated in vitro cultured cells with 100UI/mL of hCG every week for 4 weeks and we showed that both patients’ gubernacular cells increased their proliferation after hormonal stimuli. Interestingly, when stimulated, the cells of patient #4 showed a significantly higher proliferation in comparison to the cells of patient #15, confirming the different temporal windows within which the two cell types started to exponentially grow (Table 1).

This study represents the first step toward a broader in vitro study and potential future clinical validations of an hCG-based therapeutic approach for patients affected by testicular torsion. However, some limits may be reported: (i) The number of patients would be implemented in the future to further corroborate the obtained results. However, as a preliminary study, our outcome was to evaluate the effectiveness of hormonal treatment on some representative cases and it is necessary to take into account that in vitro evaluations can hardly be performed on a large set of patients. (ii) A surgical biopsy must be performed correctly; however, due to torsion, the difference between the gubernaculum, scrotal fat, etc., was not always clear, also considering the edema and the scrotal hematoma. (iii) In this study, we considered as a control patient a 2-year-old cryptorchid child whose tissue had been taken from our tissue biobank and processed following the same protocol of the other patients; the response to the hCG treatment of the control patient was considered representative of a control group presenting a healthy testicle and having a good rate of response to hormonal stimulation. (iv) A future step would be to compare the in vitro response to the treatment with clinical information that would be obtained by analyzing testicular trophism and vascularization at the end of therapeutic treatment on the same patient. (v) It would be necessary to delineate a cut-off of therapy responder patients in order to propose orchiectomy after treatment.

Clinically, to decide whether an injured testicle should be kept or removed during surgery, it will be fundamental to recognize the quality of the testicle in order to predict how it (and the gubernaculum) will respond to therapy. Finally, it is noteworthy that the data on in vitro proliferation analysis were obtained by treating the cells with a single standardized dosage. Therefore, a future goal would be to evaluate how the cells of each patient proliferate under different conditions of treatment with chorionic gonadotropin and to identify the dose and timing at which the gubernaculum of a given patient responds more. This information will permit proposing a tailored therapy for each patient.

## 5. Conclusions

In this preliminary study, we demonstrate for the first time that a surgical biopsy of a small piece of the gubernaculum from pediatric patients affected by testicular torsion can grow in culture when the testis is not too damaged, paving the way for future experiments aimed at evaluating in vitro the vital state of the testes and predicting in advance whether treatment with chorionic gonadotropin may improve the testicle condition and function in a personalized way.

## Figures and Tables

**Table 1:** Clinical and biological evaluations of the analyzed patients and patient derived cells.

Patient	Age	Clinical Evaluation	Biological Evaluation (Days Necessary for Cells to Exponentially Grow)
#4	12 yrs	Testicle torsion—preservation of testicle after derotation	7 days
#12	14 yrs	Testicle torsion—removal of testicle due to necrosis	N/A
#15	14 yrs	Morgagni hydatid torsion and epididymitis	30 days

## Data Availability

Data available on request to corresponding authors.

## Abbreviations

hCG	human chorionic gonadotropin
LH	luteinizing hormone
LHR	luteinizing hormone receptor
AR	androgen receptor
